# Clinical outcomes of *ROS1*-positive non-small cell lung cancer with limited access to *ROS1*-tyrosine kinase inhibitors (TKIs): experience from an Indian tertiary referral centre

**DOI:** 10.3332/ecancer.2024.1654

**Published:** 2024-01-15

**Authors:** Goutam Santosh Panda, Vanita Noronha, Vijay Patil, Amit Joshi, Nandini Menon, Rajiv Kumar, Trupti Pai, Omshree Shetty, Amit Janu, Nivedita Chakrabarty, Nilendu Purandare, Sayak Dey, Kumar Prabhash

**Affiliations:** Tata Memorial Hospital, Homi Bhabha National Institute (HBNI), Dr E Borges Road, Parel, Mumbai 400 012, India

**Keywords:** ROS1, non-small cell lung cancer, TKI, overall survival, progression free survival

## Abstract

**Introduction:**

*ROS1* as a driver mutation is observed in approximately 1%–2% of all non-small cell lung cancer (NSCLC). Given its rarity, we share our experience regarding *ROS1*-positive NSCLC including the access to *ROS1* tyrosine kinase inhibitors (TKIs) in a low-middle income country like India.

**Methods:**

It is a retrospective analysis of *ROS1*-positive NSCLC patients registered between January 2015 to December 2021 for demographics, treatment patterns and outcomes i.e., overall survival (OS) and progression free survival (PFS).

**Results:**

Baseline characteristics were available for 70 patients of 78 patients positive for *ROS1* by fluorescent *in situ* hybridisation. Median age at presentation was 52 years, 39 (55.7%) were males, most (51, 72.86%) were non-smokers and ten patients (14.3%) had poor Eastern Cooperative Oncology Group (ECOG) performance status (PS) i.e., PS >2 at presentation. A total of 67 patients receiving cancer directed therapy were analysed for survival. The first line (1L) therapies included – *ROS1* TKIs in 38, chemotherapy in 20, epidermal growth factor receptor TKI in eight and chemotherapy-bevacizumab in one only. *ROS1* TKI was provided to 20 patients as part of an assistance programme. The median OS for patients who received *ROS1* TKI was not attained (95% CI 37.85–NA), while it was 8.11 (95% CI 6.31–NA) months for those who did not (HR-0.1673). The median PFS for the 1L *ROS1* TKI compared to the no-TKI group was 27.07 (95% CI 24.28–NA) months versus 5.78 (95% CI 3.42–12) months (HR: 0.2047). Poor ECOG PS at presentation was the only independent prognosticator for survival.

**Conclusion:**

Using *ROS1* TKI improves clinical outcomes in all-comers though statistically not significant. To further improve outcomes, future trials should pay special attention to patients with poor PS and find a way to increase the current limited access to TKI.

## Introduction

*ROS1* is considered to be an oncogenic driver mutation in non-small cell lung cancer (NSCLC) and its rearrangements constitute a unique molecular class of NSCLC accounting for 1%–2% of all NSCLC [1, 2]. The homology of amino acids (approximately 77%) in the kinase domain of *ALK* and *ROS1* explains the activity of crizotinib, an *ALK* tyrosine kinase inhibitors (TKI) against *ROS1* [3, 4]. Crizotinib gained Food and Drug Administration (FDA) approval after the PROFILE 1001 study, a single-arm study of 50 patients, which demonstrated an overall response rate (ORR) of 72% and a median progression-free survival (PFS) of 19.2 months [5]. Also, these patients are known to have a durable response to pemetrexed (PFS up to 9 months) [6, 7] compared to non-squamous NSCLC with no targetable mutations (PFS 4 months) [8]. Also, another retrospective study of 77 patients has demonstrated that pemetrexed-platinum doublet followed by pemetrexed maintenance leads to inferior PFS, but not overall survival (OS) compared to crizotinib in first line (1L) [9]. In a low-middle income country (LMIC) like India most patients can't afford TKI and opt for systemic chemotherapy. We report the demographics, treatment patterns including access to *ROS1* TKIs and clinical outcomes of these patients.

## Methods

This is a retrospective analysis of all advanced NSCLC patients who were >18 years at the age of diagnosis and had *ROS1* rearrangement detected by fluorescent *in situ* hybridisation (FISH). Patients registered at Tata Memorial Hospital, Mumbai from 01/05/2015 to 31/12/2021 were included. Prior history of malignancy or treatment for malignancy, synchronous malignancies were not exclusion criteria. However, those without any clinical details were excluded from analysis. The patients’ clinico-pathological details were obtained from records maintained in the department of pathology and medical oncology. Clinical data from the electronic medical records were also used. Central nervous system (CNS) imaging and cerebro-spinal fluid (CSF) study were considered for symptomatic patients only. However CNS cross-sectional imaging was available for some asymptomatic patients who underwent a whole body fluorodeoxyglucose positron emission-computed tomography. No financial support was utilised for this study. Prior approval of the Institutional ethics committee was taken.

Response to treatment was documented according to response evaluation criteria in solid tumours 1.1. Also Common Terminology Criteria for Adverse Events (CTCAE) v4 was used for recording the grade 3 or higher toxicities. ORR and disease control rate (DCR) were calculated as the percentage of total patients who achieved at least partial response and stable disease as the best response, respectively on therapy.

### Statistical analysis

All the data were entered in an Excel sheet and R Studio v1.4.1743-4 was used for statistical analysis. We used survival package V3.3-1 to obtain survival estimates using Kaplan Meier method for survival analysis. Median follow up was calculated using reverse Kaplan Meier. OS was calculated from the date of start of *ROS1* TKI (if ever received in 1L) or therapies other than *ROS1* TKI (if never received *ROS1* TKI in 1L) to the last date of follow up or date of death due to any cause, whichever occurred earlier. PFS was calculated as the time interval between the start of *ROS1* TKI (if received in 1L) or therapies other than *ROS1* TKI (if never received *ROS1* TKI in 1L) and the date of progression, death or last follow up, whichever occurred earlier. Factors that might have an impact on survival were subjected to univariate analysis and later Cox’s multivariate analysis was carried out for factors significant on univariate analysis.

## Results

### Baseline patient and tumour characteristics

**Figure 1.**
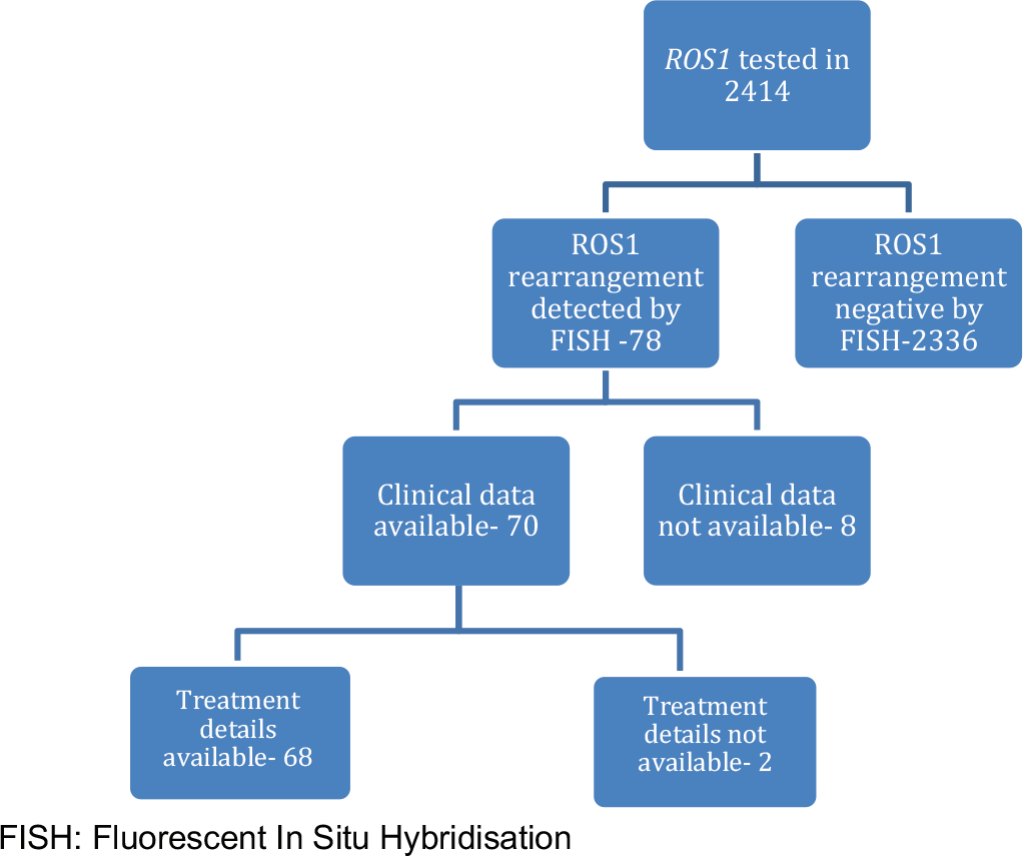
Schematic representation of number of patients who were tested and number of patients with baseline available parameters. FISH: Fluorescent *in situ* hybridisation.

*ROS1* was tested in 2,414 NSCLC patients during this time period. Of these, 78 patients were detected to have *ROS1* rearrangement by FISH. Because of the non-availability of clinical data in 8 cases, the baseline characteristics of the remaining 70 patients were analysed. Two patients defaulted before the start of any treatment at our institute and were not contactable also (Figure 1). Median age at presentation was 52 years, 19 (27.1%) were smokers, 39 (55.7%) were male. Comorbidity was noticed in 21 (30%) patients with hypertension being the most common one (Table 1). Of these *ROS1*-positive cases, the most common site of metastasis was lungs (49, 70%) followed by non-regional lymph nodes (32, 45.71%). CNS involvement at the time of diagnosis of metastatic disease was noted in 13 (18.57%) patients. Additional genetic aberration in the form of epidermal growth factor receptor (EGFR) positivity was seen in only one patient. Family history of malignancy was present in 8 (11.43%) patients. Baseline next generation sequencing (NGS) was available for only three patients and fusion partners detected were cluster of differentiation (CD) 74 in two cases and SLC34A2 in one case.

### 1L treatment

Of these 70 patients, treatment details were available for 68 patients. Moreover, one patient defaulted initially, only to turn up after 2 months of diagnosis in very poor general condition and death. Hence for survival analysis, 67 patients were considered. Only two patients were non-metastatic at presentation and were initially treated with curative intent i.e., chemo-radiation. Subsequently, on progression, these patients were treated in the line of metastatic disease. Treatment for metastatic disease included chemotherapy (pemetrexed-platinum), *ROS1* TKI, chemo-immunotherapy and others (Table 2). Patients who were in poor general condition and were deemed unfit for systemic chemotherapy were offered EGFR first- generation TKI (erlotinib)-7 cases. Also, erlotinib was considered for one patient who denied chemotherapy before the availability of *ROS1* FISH and was lost to follow-up after first follow up visit. Before the availability of *ROS1* FISH report, 25 patients received chemotherapy +/− anti-angiogenic agent +/− immunotherapy first and *ROS1* TKI was started upon availability of FISH report. The median number of chemotherapy cycles before starting *ROS1* TKI in such cases was two. Twenty patients received *ROS1* TKI (crizotinib, entrectinib) through a patient assistance programme.

One patient was treated with gefitinib + crizotinib for dual positivity i.e., EGFR sensitising mutation along with *ROS1* rearrangement. This patient was 54 years old, diabetic and hypertensive male who presented to the Outpatient Department with breathlessness. He was evaluated and diagnosed as metastatic adenocarcinoma lung with pleural effusion. The pleural effusion was drained using an intercostal drainage tube. EGFR exon 19 deletion and *ROS1* positivity by FISH were found through molecular testing. Following a discussion in the internal molecular tumour board, he was started on crizotinib in addition to gefitinib because the patient could not afford osimertinib. He developed grade 2 papulopustular rash. It was treated conservatively by temporarily discontinuing TKIs and oral antibiotics.

### ORR and DCR

The ORR for patients receiving *ROS1* TKI was 85.29% and the DCR was 100%. The ORR and DCR for those patients who received chemotherapy initially and later switched to *ROS1* TKI were 78.95% and 100%, respectively. Patients who received chemotherapy only had ORR and DCR of 55% and 90%, respectively. One patient denied intravenous chemotherapy; hence erlotinib was started on a compassionate basis and stable disease after 3 months was noted. Table 3 describes ORR and DCR with different therapies

### Treatment post-progression on 1L

Of all 67 patients analysed for survival, 43 had disease progression either clinically and/or radiologically. Of these 43 patients, only 21 received second-line therapy remaining 22 did not receive second- line therapy because of either poor general condition (12 patients) or defaulting patients (10 patients). The second line therapies included: *ROS1* TKI in 8 (crizotinib: 3 patients, lorlatinib :5 patients), chemotherapy + crizotinib in 3, chemotherapy in 7, chemo-bevacizumab in 2, gefitinib in 1. For five patients progressing in the brain, *ROS1* TKI was continued in three after whole brain radiotherapy while for the remaining two *ROS1* TKI was changed to lorlatinib from crizotinib. Of all the patients progressing on the 1L therapy, repeat biopsy was done for only five patients and NGS on repeat biopsy was carried out for two patients as remaining three patients had either financial issues or inadequate tumour tissue.

The patient with dual oncogene (EGFR and *ROS1* positivity) developed oligopression in the mediastinal nodes with PFS of 11 months on gefitinib plus crizotinib. He was offered radiotherapy for oligoprogression. A repeat biopsy revealed adenocarcinoma and NGS was ordered. NGS revealed SLC34A2 and *ROS1* fusion with a read count of 5,640 along with a tier II (likely pathogenic) missense mutation in exon 12 of *RET* gene with a variant frequency of 4.10. After 2 months of radiotherapy, the patient developed radiation pneumonitis grade 2. TKIs were stopped and steroids were started to treat radiation pneumonitis. The case was reviewed in molecular tumour board and chemotherapy (pemetrexed plus carboplatin) was initiated because the patient could not afford lorlatinib. For the past 3 months, the patient has been undergoing chemotherapy.

#### Sites of progression on ROS1 TKI

Of 38 patients, 16 (42.11%) progressed on *ROS1* TKI. The most common sites of progression were CNS and lungs (noticed in five each). Cross-sectional imaging was unavailable for four patients who had progressed clinically.

### Clinical outcomes

#### Entire cohort

The median follow up was 14.5 (95% CI 13.0–41.9) months in surviving *ROS1* positive NSCLC patients.

**Figure 2.**
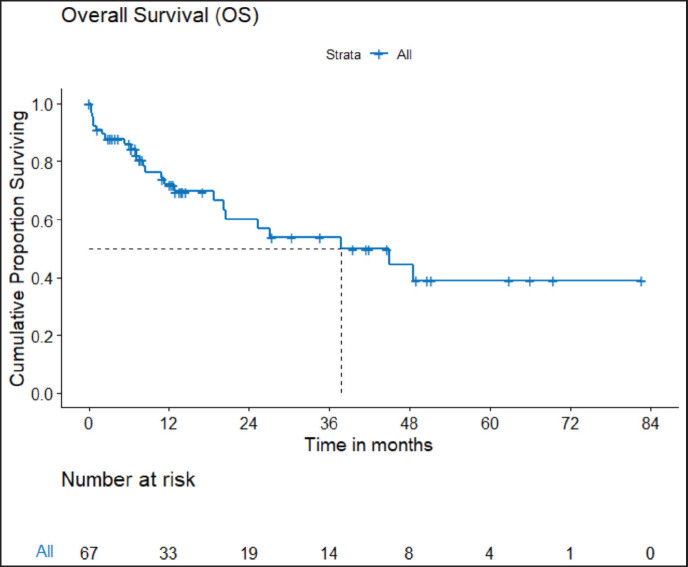
OS of *ROS1*-positive NSCLC.

**Figure 3.**
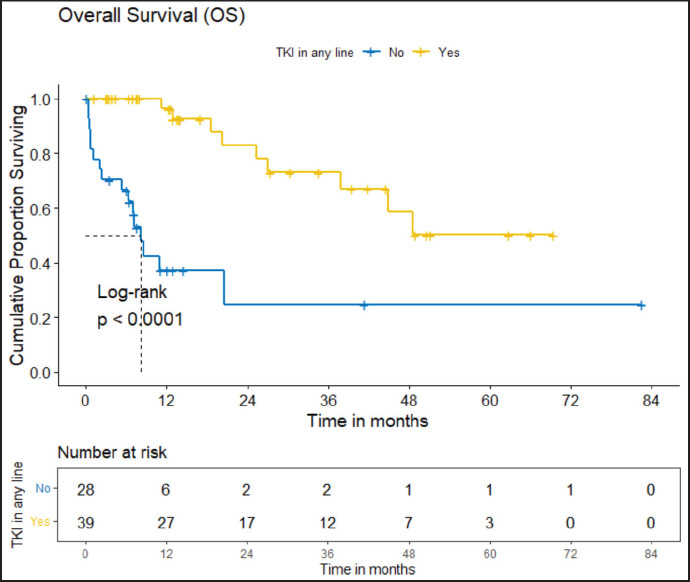
OS of the cohort who received *ROS1* TKI in any line versus never received any *ROS1* TKI.

- PFS: The median PFS was 13 (95% CI 9.92–26.1) months. The estimated PFS at 3 and 5 years are 26.4% (95% CI 16.15%–43.2%) and 15.4% (95% CI 6.74%–35.2%), respectively.
- OS: There were a total 25 deaths with 1 death attributable to the chemotherapy-toxicity. The median OS was 37.9 (95% CI 20.6–NA) months. The projected 3 and 5 years OS are 53.8% (95% CI 40.3%–71.8%) and 38.8% (95% CI 24.1%–62.5%), respectively. Figure 2 depicts the OS in the entire cohort while Figure 3 shows the differential outcome with the use of *ROS1* TKI.

**Figure 4.**
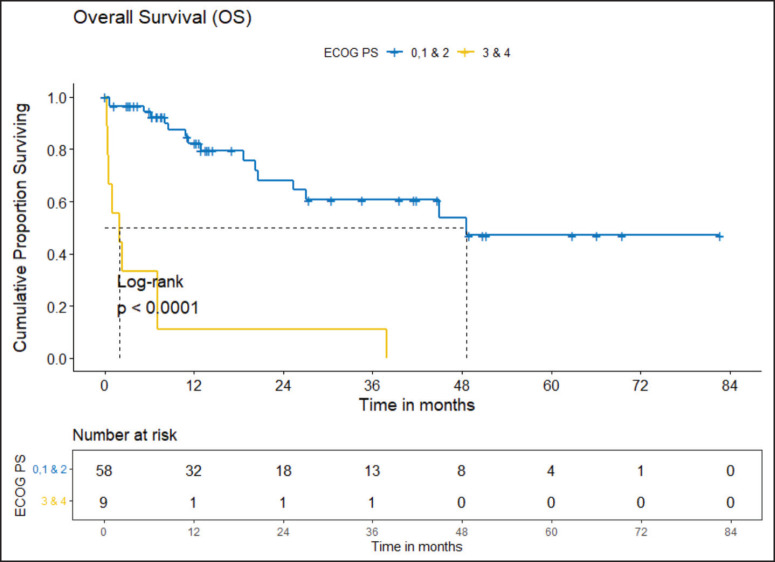
OS comparison of *ROS1*-positive cohort according to ECOG PS. (ECOG PS 0, 1, 2 versus ECOG PS 3, 4).

**Figure 5.**
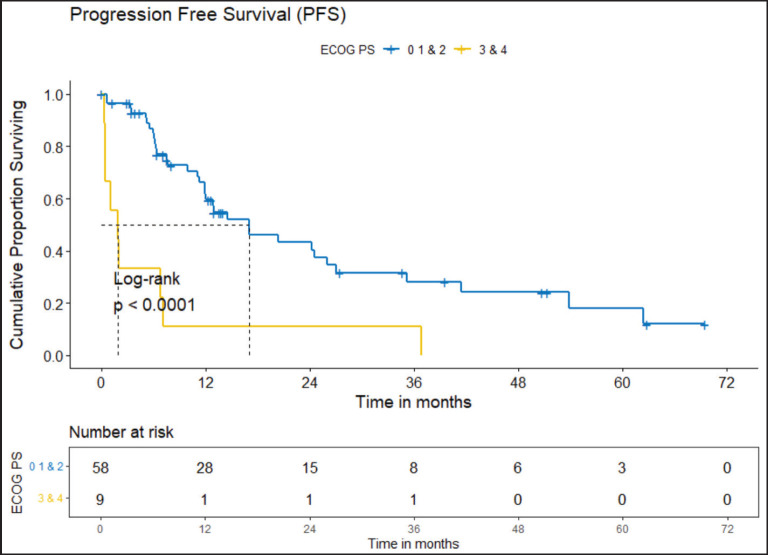
PFS comparison of *ROS1*-positive cohort according to ECOG PS. (ECOG PS 0, 1, 2 versus ECOG PS 3, 4).

The clinical outcomes for patients who received *ROS1* TKI in 1L versus chemotherapy are presented in Table 4. Also, Figures 4 and 5 depict clinical outcomes (OS and PFS) of the entire cohort according to Eastern Cooperative Oncology Group (ECOG) performance status (PS). The median OS of patients who received *ROS1* TKI in 1L versus who did not receive were 48.59 (95% CI 37.85–NA) months versus 8.11 (95% CI 6.31–NA) months (*p* = 0.0001) while the median PFS for the above groups were 27.07 (95% CI 24.28–NA) months versus 5.78 (95% CI 3.42–12) months (*p* = 0.0001), respectively.

#### Adverse events

Patients tolerated the treatment well, with grade ≥3 adverse events in a total of four patients (4/67, 5.97%). The recorded adverse events were grade 3 anaemia (3 cases), grade 3 transaminitis and grade 4 thrombocytopenia and grade 3 skin toxicity in one each.

#### Prognostic factors

**Figure 6.**
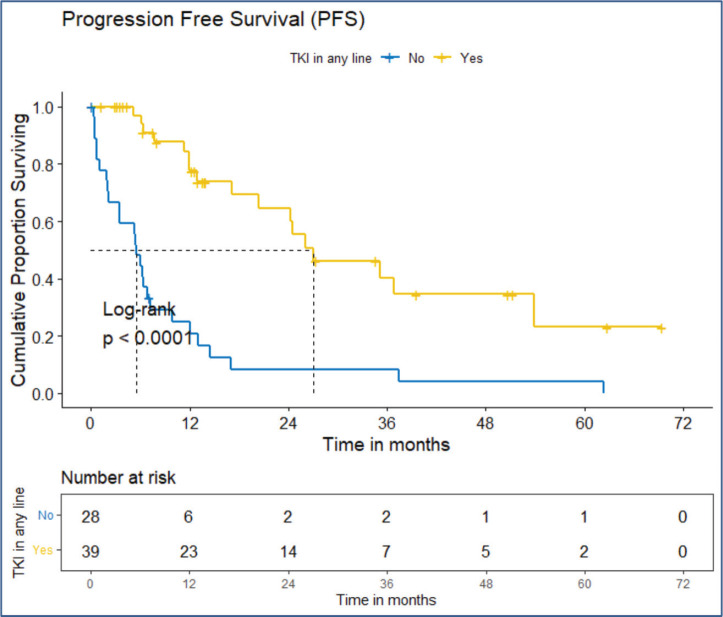
PFS of the *ROS1*-positive cohort who received ROS1 TKI in any line versus never received any *ROS1* TKI.

The factors significant in univariate analysis are tabulated in Tables 5 and 6. Multivariate analysis revealed only poor ECOG PS at presentation i.e., ECOG PS >2 to be independent predictors of inferior OS (HR-7.685, 95% CI 2.8429–20.773, *p* < 0.0001) and PFS (HR-3.0211, 95% CI 1.33674–6.828, *p* = 0.00787) as depicted in Figures 4 and 5. The median PFS and OS for patients with ECOG PS >2 at presentation were 1.94 (95% CI 0.49–NA) months and 2 (95% CI 0.49–NA) months, respectively. Figure 2 depicts the outcomes in patients with the use of *ROS1* TKI. As seen in Figure 6, the use of *ROS1* TKI improved PFS. Though the use of *ROS1* TKI was associated with statistically significant improved survival in univariate analysis, the improvement was insignificant on multivariate analysis. There was no statistically significant difference in the survival of patients who received interim chemotherapy awaiting *ROS1* FISH report.

## Discussion

We had screened a total of 2,414 patients for *ROS1* and 78 were positive by FISH, accounting to 3.23%. Our previous report also had an increased incidence of *ROS1* positivity (4%) compared to 1%–2% as per literature [3, 10]. Another study from North India also demonstrates a higher incidence (2.8%) of *ROS1* positive NSCLC [11]. The higher incidence of *ROS1* positive NSCLC might be because of our initial institutional practice of asking for *ROS1* FISH for only EGFR negative and ALK rearrangement negative cohort or might be reflective of actual higher incidence of *ROS1* rearranged NSCLC in India. Larger multicentre studies are needed to know the exact incidence so that we can modify our management policy accordingly. Also, a larger number of non-smokers was noticed in our study, similar to other studies in oncogene-addicted NSCLC [12–14]. The median age at diagnosis (52 years) in our cohort was in line with other reports including our previous observation by Joshi *et al* [15] and the landmark PROFILE 1001 study [5, 16–18]. However, some other authors have reported the median age to be slightly higher [19, 20]. Almost 20% of our patients had CNS involvement at presentation, which is consistent with other studies that show an increased incidence of CNS involvement in these oncogene-addicted patients [21]. Another important feature in demographics in developing countries is that a sizable number of patients presenting with poor PS. We have included all comers in this study with ten patients presenting as ECOG PS >2. Most trials have excluded these patients as the survival is poor. We have demonstrated and published the benefit of initial reduced dose chemotherapy in small cell lung cancer patients with poor ECOG PS at presentation [22].

India being an LMIC, not many patients can afford *ROS1* TKI. However, patients do get some financial support from several non-governmental organisations. More than half (52.6%) of the 38 patients who received *ROS1* TKI in the 1L did so through an assistance programme. As these drugs such as crizotinib, ceritinib and entrectinib are not pocket friendly for most of our patients, in addition to assistance from the government, we should look for cheaper solutions in the form of developing other indigenous effective molecules or generic drugs. Since erlotinib is well tolerated and EGFR mutations are more common (23%–30.03%) [23, 24], it was offered to patients who were in poor general condition and were deemed unfit for systemic chemotherapy.

In our study, patients had a median PFS and OS of 13 and 37.9 months, respectively. In the seminal PROFILE 1001 study, at a median follow up of 62.6 months, the median PFS and OS were 19.3 months (95% CI, 15.2–39.1) and 51.4 months (95% CI, 29.3 to not reached), respectively [25]. As all patients were treated with *ROS1* TKI in the PROFILE 1001 trial, the outcome is expected to be better than ours. When compared to the PROFILE 1001 study, patients who received *ROS1* TKI in our study had a better PFS but a similar OS. The longer PFS in the *ROS1* TKI group in our study could be due to a possible different fusion partner, a smaller number of patients receiving *ROS1* TKI and/or a shorter follow-up period. Patients having certain fusion variants such as CD 74 have better PFS (median PFS of 20.1 months) compared to others patients harbouring non-CD 74 fusion variants (median PFS 12.0 months) [9, 26]. In the PROFILE 1001 study, nearly half of the patients (46%) had received two or more prior metastatic/advanced regimens, which may explain the lower PFS. Another phase 2 study by Landi *et al* [27] has reported the median PFS to be 22.8 months using crizotinib in patients who have received at least one line of chemotherapy. We noticed a similar OS of our cohort treated with *ROS1* TKI when compared to the literature [18, 25]. Another reason for relatively higher PFS in our cohort receiving *ROS1* TKI is the continuation of TKI for asymptomatic patients who developed small new lung nodules that meet the criteria for progressive disease and would have qualified as a progressive disease in clinical trials. Only one patient in our cohort who did not receive *ROS1* TKI in the 1L received it in the second-line. As a result, we are unable to compare the efficacy of *ROS1* TKI in 1L to later-lines therapy. Shen *et al* [9] have demonstrated that using crizotinib as a second or later-line therapy does not impair survival if it cannot be administered as an 1L therapy. In our study, the 1L *ROS1* TKIs were ceritinib in 4 patients, entrectinib in 4, and crizotinib in 33. As a result, comparing the efficacy of different *ROS1* TKIs in the 1L is inappropriate.

Our patients on *ROS1* TKI had a median PFS and OS of 27.07 months and 48.59 months compared to 6.87 and 10.9 months on chemotherapy. And except for 1 patient, none of the 20 patients who received chemo in 1L received *ROS1* TKI later on. This establishes the importance of precision oncology in the current era and emphasises the use of targeted therapy. The effectiveness of *ROS1* targeted therapy (TKI) is well known [9, 25, 28]. Also, the response rates observed in our cohort are in sync with other studies [9, 28]. 

It is well known that ECOG PS at presentation is a strong prognostic factor in lung cancer [29, 30]. In our cohort, the median PFS and OS for patients with poor PS were 1.94 and 2 months, respectively. Patients who received *ROS1* TKI had better clinical outcomes. Though the outcomes with *ROS1* TKI were numerically superior compared to other systemic therapies, it was statistically not significant. This might be due to a small sample size, short follow up.

Though there is no level 1 evidence due to the rarity of the tumour with dual EGFR and *ROS1* positivity, literature supports the use of TKI rather than chemotherapy in such cases [31, 32].

## Limitations

Due to the fact that this is a single-institution study, the number of patients are less and the follow-up period is brief. We didn't have *ROS1* fusion partners as the FISH test utilised a break apart probe (except for three cases where baseline NGS was done). Being retrospective in nature, it also has all the shortcomings of a retrospective study.

## Conclusion

The incidence of *ROS1* rearranged NSCLC appears to be higher than what has been reported in the literature. In India, access to the *ROS1* TKI is currently limited. The use of *ROS1* TKI improves the outcome. Poor ECOG PS at presentation is associated with poor outcomes. To further improve outcomes, future trials should pay special attention to patients with poor PS and find a way for increased access to TKI.

## Conflicts of interest

The authors declare that they have no known competing financial interests or personal relationships that could have appeared to influence the work reported in this paper.

## Funding

No research support was received for this study.

## Institutional ethics committee permission

Obtained.

## Figures and Tables

**Table 1.** Baseline parameters of patients with *ROS1* positive NSCLC.

Patients’ characteristics	*n* = 70, (%)
Age in years, Median (Range)	52 (26–78)
Gender Male Female	39 (55.70%) 31 (44.30%)
ECOG PS 0–1 2 >2	45 (64.3%) 15 (21.4%) 10 (14.3%)
Comorbidity Yes Hypertension Diabetes mellitus Chronic kidney disease Ischemic heart disease No	21 (30.0%) 14 (20.0%) 12 (17.14%) 3 (4.29%) 2 (8.86%) 49 (70.0%)
Histology Adenocarcinoma	70 (100.0%)
Smoking status Never-smoker Ever-smoker	51 (72.86%) 19 (27.14%)
Family history of malignancy Yes No Not documented	8 (11.43%) 41 (58.57%) 21 (30%)
Baseline AJCC stage I II III IV	0 (0%) 0 (0%) 2 (2.86%) 68 (97.14%)
Initial sites of metastasis Lungs (parenchymal/(pleural deposits and effusion)) Bone Liver CNS Adrenal gland Non-regional lymph node Others	49 (70.0%) (36 (51.4%)/31 (44.3%)) 20 (28.57%) 10 (14.3%) 13 (18.57%) 7 (10.0%) 32 (45.71%) 4 (5.71%)

**Table 2.** 1L treatment of patients with *ROS1* positive NSCLC.

1L treatment for metastatic disease	*n* = 68 (%)
A. *ROS1* TKI	38 (55.88%)
1. Upfront *ROS1* TKI a. Crizotinib b. Entrectinib c. Ceritinib 2. Chemotherapy followed by *ROS1* TKI 3. Chemo-immunotherapy followed by *ROS1* TKI 4. Chemo-bevacizumab followed by *ROS1* TKI 5. *EGFR* TKI + *ROS1* TKI	12 (17.65%) 9 (13.24%) 1 (1.47%) 2 (2.94%) 21 (30.88%) 3(4.41%) 1 (1.47%) 1 (1.47%)
B. Other than *ROS1* TKI	30 (44.12%)
1. Chemotherapy only (Pemetrexed-carboplatin) 2. Chemo-bevacizumab 3. *EGFR* TKI 4. No treatment	20 (29.41%) 1 (1.47%) 8 (11.76%) 1 (1.47%)

**Table 3.** ORR and DCR with various treatments for patient with *ROS1*-positive NSCLC.

Treatment (*n* = 68)	ORR (%)	DCR (%)	Not evaluated
A. Received *ROS1* TKI in 1L (*n* = 38)^b^	29/38 (76.32%)	34/38 (89.47%)	4/38 (10.53%)
1. *ROS1* TKI alone (*n* = 12)^b^	10/12 (83.33%)	10/12 (83.33%)	2/12 (16.67%)
a. Crizotinib (*n* = 9)	9/9^a^ (100%)	9/9^a^ (100%)	–
b. Entrectinib (*n* = 1)^b^	–	–	1/1 (100%)
c. Ceritinib (*n* = 2)^b^	1/2 (50%)	1**/**2(50%)	1/2(50%)
2. Chemotherapy followed by *ROS1* TKI (*n* = 21)^b^	15/21 (71.43%)	19/21 (90.48%)	2/21 (9.52%)
3. Chemo-immunotherapy followed by *ROS1* TKI (*n* = 3)	2/3 (66.67%)	3/3 (100%)	–
4. EGFR TKI + *ROS1* TKI (*n* = 1)	1/1 (100%)	1/1 (100%)	–
5. Chemo-bevacizumab followed by *ROS1* TKI (*n* = 1)	1/1 (100%)	1/1 (100%)	–
B. Systemic therapies other than *ROS1* TKI in 1L	12/29 (41.38%)	20/29 (68.97%)	
1. Chemotherapy only (Pemetrexed-carboplatin)^c^	11/20 (55%)	18/20 (90%)	–
2. Pemetrexed-carboplatin-bevacizumab	1/1 (100%)	1/1 (100%)	–
3. EGFR TKI^d^	0/8 (0%)	1/8 (12.5%)	–
C. No treatment	0/1	0/1	

a One patient achieved complete metabolic response on crizotinib

b Not evaluated radiologically: 4 = 1 patient on entrectinib and ceritinib each +2 patients on crizotinib after initial chemotherapy

c Two patients progressed clinically. No response scan was done

d One patient had stable disease on CT scan and defaulted post first follow up while seven others progressed clinically and no response scan was done

*EGFR* = Epidermal growth factor receptor; TKI= Tyrosine kinase inhibitor

**Table 4.** Clinical outcomes of patients who received ROS1 TKI versus those who received chemotherapy.

Cohort (*n* = 58)	Median follow up (95% CI) in months	Median PFS (95% CI) in months	3 years PFS/5 years PFS	Median OS(95% CI ) in months	3 years OS/5 years OS
*ROS1* TKI in 1L (*n* = 38)	27.4 (95% CI 13.0–50.8)	27.07 (95% CI 24.28–NA)	41.8%/23.9%	48.59 (95% CI 37.85–NA)	71.8%/46.6%
Chemotherapy (*n* = 20)	14.5 (95% CI 12.1–NA)	6.87 (95% CI 5.55–14.5)	10.53%/5.26%	10.9 (95% CI 7.16–NA)	36.7%/36.7%

**Table 5.** Significant prognostic factors on univariate analysis for OS.

		Median	0.95LCL–0.95UCL	*p*	HR	0.95LCL–0.95UCL
TKI used in 1L of therapy	No	8.11	6.31–NA	0.0001		
	Yes	48.59	37.85–NA		0.2168	0.0941–0.4997
TKI used in any line of therapy	NO	8.11	6.31–NA	0.0001		
	Yes	NA	37.85–NA		0.1673	0.07131–0.3923
ECOG PS at presentation	0–2	48.6	25.3–NA	0.0001		
	3–4	2	0.49–NA		9.935	4.192–23.55

**Table 6.** Significant prognostic factors on univariate analysis for PFS.

		Median	0.95LCL–0.95UCL	*p*	HR	0.95LCL–0.95UCL
TKI used in 1L line of therapy	No	5.78	3.42–12	0.0001		
	Yes	27.07	24.28–NA		0.2047	0.1084–0.3866
ECOG PS at presentation	0–2	17.05	11.96–35.1	0.0001		
	3–4	1.94	0.49–NA		4.323	2.032–9.197
Serum albumin at presentation	≤3.9 g/dL	11.9	6.87–24.5	0.044		
	>3.9 g/dL				2.327	1–5.414
Serum alkaline phospahatase	≤109 U/L	24.28	17.05–NA	0.0026		
	>109 U/L	9.92	6.37–24.5		2.669	1.377–5.174

## References

[1] Bergethon K, Shaw AT, Ou SHI (2012). ROS1 rearrangements define a unique molecular class of lung cancers. J Clin Oncol.

[2] Takeuchi K, Soda M, Togashi Y (2012). RET, ROS1 and ALK fusions in lung cancer. Nat Med.

[3] Gainor JF, Shaw AT (2013). Novel targets in non-small cell lung cancer: ROS1 and RET fusions. Oncologist.

[4] Huber KVM, Salah E, Radic B (2014). Stereospecific targeting of MTH1 by (S)-crizotinib as an anticancer strategy. Nature.

[5] Shaw AT, Ou SHI, Bang YJ (2014). Crizotinib in ROS1-rearranged non-small-cell lung cancer. N Engl J Med.

[6] Song Z, Su H, Zhang Y (2016). Patients with ROS1 rearrangement-positive non-small-cell lung cancer benefit from pemetrexed-based chemotherapy. Cancer Med.

[7] Chen YF, Hsieh MS, Wu SG (2016). Efficacy of pemetrexed-based chemotherapy in patients with ROS1 fusion-positive lung adenocarcinoma compared with in patients harboring other driver mutations in East Asian populations. J Thorac Oncol.

[8] Paz-Ares L, Marinis F, Dediu M (2012). Maintenance therapy with pemetrexed plus best supportive care versus placebo plus best supportive care after induction therapy with pemetrexed plus cisplatin for advanced non-squamous non-small-cell lung cancer (PARAMOUNT): a double-blind, phase 3, randomised controlled trial. Lancet Oncol.

[9] Shen L, Qiang T, Li Z (2020). First-line crizotinib versus platinum-pemetrexed chemotherapy in patients with advanced ROS1-rearranged non-small-cell lung cancer. Cancer Med.

[10] Chen YF, Hsieh MS, Wu SG (2014). Clinical and the prognostic characteristics of lung adenocarcinoma patients with ROS1 fusion in comparison with other driver mutations in East Asian populations. J Thorac Oncol.

[11] Mehta A, Saifi M, Batra U (2020). Incidence of ROS1-rearranged non-small-cell lung carcinoma in India and efficacy of crizotinib in lung adenocarcinoma patients. Lung Cancer.

[12] Tseng CH, Chiang CJ, Tseng JS (2017). EGFR mutation, smoking, and gender in advanced lung adenocarcinoma. Oncotarget.

[13] Noronha V, Prabhash K, Thavamani A (2013). EGFR mutations in Indian lung cancer patients: clinical correlation and outcome to EGFR targeted therapy. PLoS One.

[14] Noronha V, Ramaswamy A, Patil VM (2016). ALK positive lung cancer: clinical profile, practice and outcomes in a developing country. PLoS One.

[15] Joshi A, Pande N, Noronha V (2019). ROS1 mutation non-small cell lung cancer-access to optimal treatment and outcomes. Ecancermedicalscience.

[16] Chen Y, Huang Y, Ning H (2019). Clinic-pathologic features and gene fusion pattern of ALK and ROS1 in non-small cell lung cancer show association with household coal combustion. Transl Cancer Res.

[17] Jurmeister P, Lenze D, Berg E (2015). Parallel screening for ALK, MET and ROS1 alterations in non-small cell lung cancer with implications for daily routine testing. Lung Cancer.

[18] Park S, Ahn BC, Lim SW (2018). Characteristics and outcome of ROS1-positive non–small cell lung cancer patients in routine clinical practice. J Thorac Oncol.

[19] Dugay F, Llamas-Gutierrez F, Gournay M (2017). Clinicopathological characteristics of ROS1- and RET-rearranged NSCLC in caucasian patients: data from a cohort of 713 non-squamous NSCLC lacking KRAS/EGFR/HER2/BRAF/PIK3CA/ALK alterations. Oncotarget.

[20] Lee J, Park CK, Yoon HK (2019). PD-L1 expression in ROS1-rearranged non-small cell lung cancer: a study using simultaneous genotypic screening of EGFR, ALK, and ROS1. Thorac Cancer.

[21] Ou SHI, Zhu VW (2019). CNS metastasis in ROS1+ NSCLC: an urgent call to action, to understand, and to overcome. Lung Cancer.

[22] Noronha V, Ravind R, Patil VM (2020). The role of chemotherapy in patients with small cell lung cancer and poor performance status. Acta Oncol.

[23] Nakra T, Mehta A, Bal A (2020). Epidermal growth factor receptor mutation status in pulmonary adenocarcinoma: multi-institutional data discussion at national conference of ‘Lung Cancer Management in Indian context’. Curr Probl Cancer.

[24] Chougule A, Prabhash K, Noronha V (2013). Frequency of EGFR mutations in 907 lung adenocarcioma patients of Indian ethnicity. PLoS One.

[25] Shaw AT, Riely GJ, Bang YJ (2019). Crizotinib in ROS1-rearranged advanced non-small-cell lung cancer (NSCLC): updated results, including overall survival, from PROFILE. Ann Oncol.

[26] Xu H, Zhang Q, Liang L (2020). Crizotinib vs platinum-based chemotherapy as first-line treatment for advanced non-small cell lung cancer with different ROS1 fusion variants. Cancer Med.

[27] Landi L, Chiari R, Tiseo M (2019). Crizotinib in MET-deregulated or ROS1-rearranged pretreated non-small cell lung cancer (METROS): a phase II, prospective, multicenter, two-arms trial. Clin Cancer Res.

[28] Wu YL, Yang JCH, Kim DW (2018). Phase II study of crizotinib in East Asian patients with ROS1-positive advanced non-small-cell lung cancer. J Clin Oncol.

[29] Jang RW, Caraiscos VB, Swami N (2014). Simple prognostic model for patients with advanced cancer based on performance status. J Oncol Pract.

[30] Kawaguchi T, Takada M, Kubo A (2010). Performance status and smoking status are independent favorable prognostic factors for survival in non-small cell lung cancer: a comprehensive analysis of 26,957 patients with NSCLC. J Thorac Oncol.

[31] Zhang Y, Wang H, Wang X (2021). Precision treatment of advanced lung adenocarcinoma with coexisting EGFR, ALK, and ROS1 mutations: a case report. Clin Lung Cancer.

[32] Gristina V, La Mantia M, Galvano A (2021). Non-small cell lung cancer harboring concurrent EGFR genomic alterations: a systematic review and critical appraisal of the double dilemma. Diagn Mol Pathol.

